# An earlier revolution: genetic and genomic analyses reveal pre-existing cultural differences leading to Neolithization

**DOI:** 10.1038/s41598-017-03717-6

**Published:** 2017-06-14

**Authors:** Michela Leonardi, Guido Barbujani, Andrea Manica

**Affiliations:** 1 Department of Life Sciences and Biotechnology, University of Ferrara, Via Borsari 44, 44121 Ferrara, Italy; 2 Department of Zoology, University of Cambridge, Downing street, CB2 3EJ Cambridge, UK; 3 Present Address: Centre for GeoGenetics, Natural History Museum of Denmark, University of Copenhagen, Oester Voldgade 5-7, DK-1350 Copenhagen, Denmark

## Abstract

Archaeological evidence shows that, in the long run, Neolitization (the transition from foraging to food production) was associated with demographic growth. We used two methods (patterns of linkage disequilibrium from whole-genome SNPs and MSMC estimates on genomes) to reconstruct the demographic profiles for respectively 64 and 24 modern-day populations with contrasting lifestyles across the Old World (sub-Saharan Africa, south-eastern Asia, Siberia). Surprisingly, in all regions, food producers had larger effective population sizes (*N*_e_) than foragers already 20 k years ago, well before the Neolithic revolution. As expected, this difference further increased ~12–10 k years ago, around or just before the onset of food production. Using paleoclimate reconstructions, we show that the early difference in *N*_e_ cannot be explained by food producers inhabiting more favorable regions. A number of mechanisms, including ancestral differences in census size, sedentism, exploitation of the natural resources, social stratification or connectivity between groups, might have led to the early differences in Ne detected in our analyses. Irrespective of the specific mechanisms involved, our results provide further evidence that long term cultural differences among populations of Palaeolithic hunter-gatherers are likely to have played an important role in the later Neolithization process.

## Introduction

The advent of food production marked a shift in human history entailing important changes in technology (e.g. mills, plant and animal domestication, use of ceramics), economy (e.g. accumulation of goods) and society (e.g. sedentism). Several lines of evidence point to this revolution leading to an increase in population density^1–4^.

Such growth is expected to have left a signature in the genomes, through a change in *N*_e_, the effective population size. Indeed, gene genealogies of expanding populations should show an excess of singletons and private alleles when compared with those of stationary populations^5^. Because recombination brings together in the same chromosome DNA tracts with different genealogies, analysis of non-recombining DNA regions (mostly in mitochondrial DNA and in the Y chromosome) is often the simplest way to investigate past changes in population size.

Genetic markers of modern day populations with different lifestyles have been compared since the late '90s, with a number of studies finding significant demographic differences between food producers and hunter-gatherers^6–10^. More recently, several studies have used mtDNA to date the beginning of effective population growth in food producers, and, unexpectedly, the increase in *N*_e_ was inferred to have started long before the Neolithic transition^11–14^. However, dating based on mtDNA can be challenging^15^, and evidence from other markers is limited. Work on the Y chromosome^16,17^ and a few autosomal loci^10,18,19^ lend some support to the notion of demographic changes predating food production, but their coverage in terms of populations is limited. The most comprehensive study to date on this topic in term of both markers and geographic coverage^18^, only included 20 autosomal loci for 16 populations, and these mostly covered Africa (n = 10), whilst the remaining 6 Eurasian populations included several highly urbanized ones (Danes, Han Chinese and Japanese) for which the more recent history could have strongly biased this kind of estimates.

Beside the suggested cultural differences (subsistence strategies, sedentism, etc.), a hypothesis that should also be taken into account is that the observed discrepancies could be the result of a differential geographic distribution of the resources^20,21^. A simple scenario might be envisaged where populations that lived in climatically more favorable areas started growing well before the advent of food production. The same favorable climate would also make the adoption of food production more likely, as it would allow for the growth of crops and a sedentary lifestyle. By contrast, populations in harsher environments might have retained hunting and gathering (and probably some level of nomadic lifestyle to exploit different resources), as the challenging climatic conditions would have made food production unfeasible; thus, we would expect these population to remained at constant sizes through time. To our knowledge, the importance of the availability of local resources to the adoption of food production has never been tested with genetic data.

**Figure 1:**
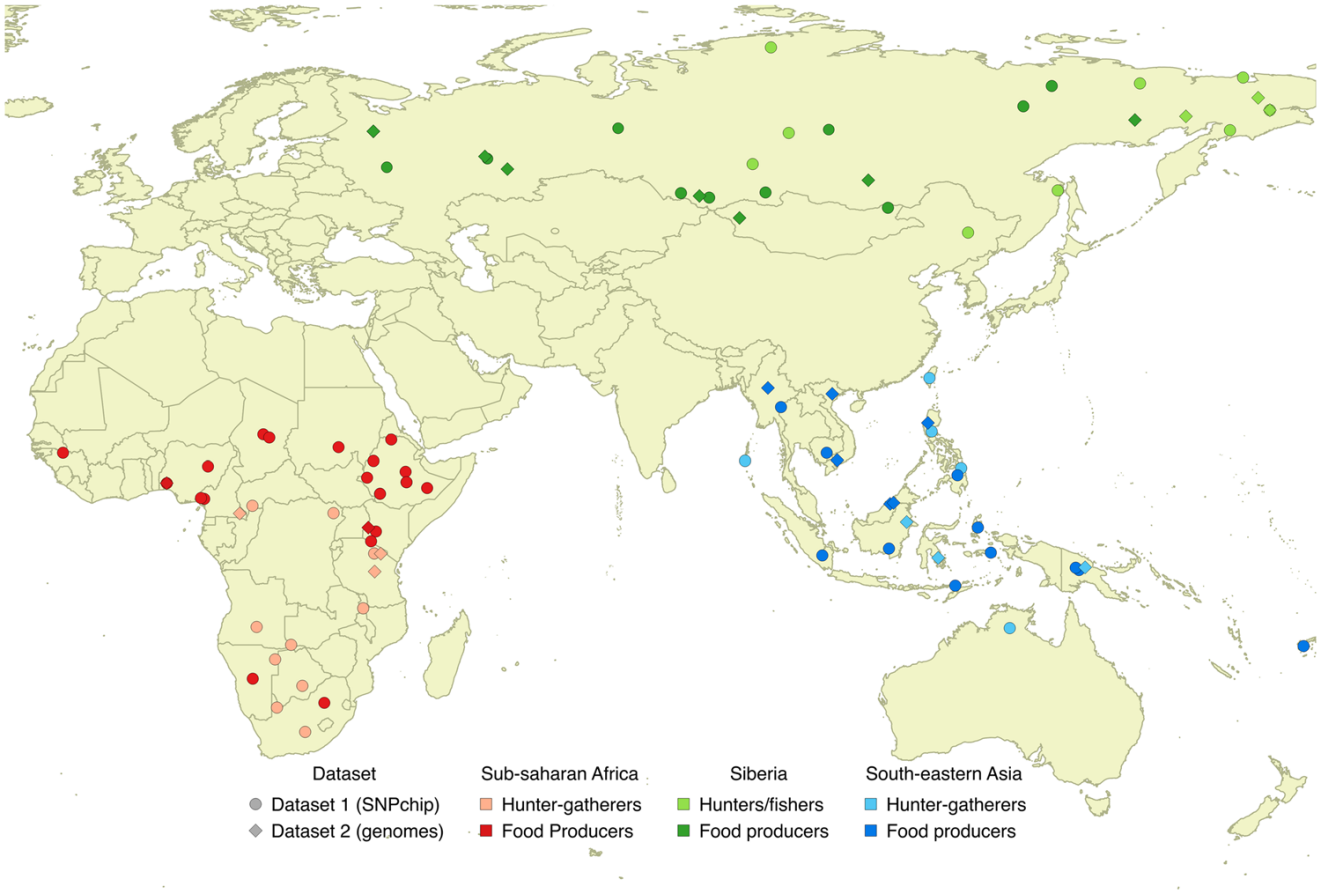
map of the populations considered in the present study. The map has been generated with the software QGIS, version 2.12.0-Lyon^60^.

In this paper, we compare the demographic trajectories of populations with different lifestyles based on two datasets (Fig. 1).

**Dataset 1**: We gathered from the literature genome-wide data (>150 k SNPs) for 64 populations^22–34^ and estimated their demographic trajectories through time using the approach based on Linkage Disequilibrium (LD) developed by Mc Evoy and colleagues^35 using the software *NeON*﻿46^. The populations were selected to avoid highly urbanized samples, and cover three major regions (sub-Saharan Africa, 28 populations, later referred to as Africa), southeastern Asia and Oceania (17 populations, later referred to as SE Asia), and Russia and Siberia (later referred to as Siberia, 19 populations).

**Dataset 2**: Pre-computed MSMC^36^ demographic estimates have been made available in a recent paper by Pagani and colleagues^37^. From this second set of data we selected 24 populations from the three already mentioned regions: Africa (5 populations), SE Asia (9 populations) and Siberia (10 populations).

We ask whether populations who turned to food production differ from hunter-gathers in their demography, and date these differences based on the estimates from the two different methods. We then proceed to test the extent to which these differences might be a direct consequence of resource availability from the surrounding environment using global paleoclimate and palaeovegetation reconstructions. Finally, to evaluate whether some demographic phenomena may generate patterns similar to those that we observed, in SE Asia we compare multiple migration scenarios that might have had a confounding effect over our inferences.

## Results

### Demography

**Figure 2:**
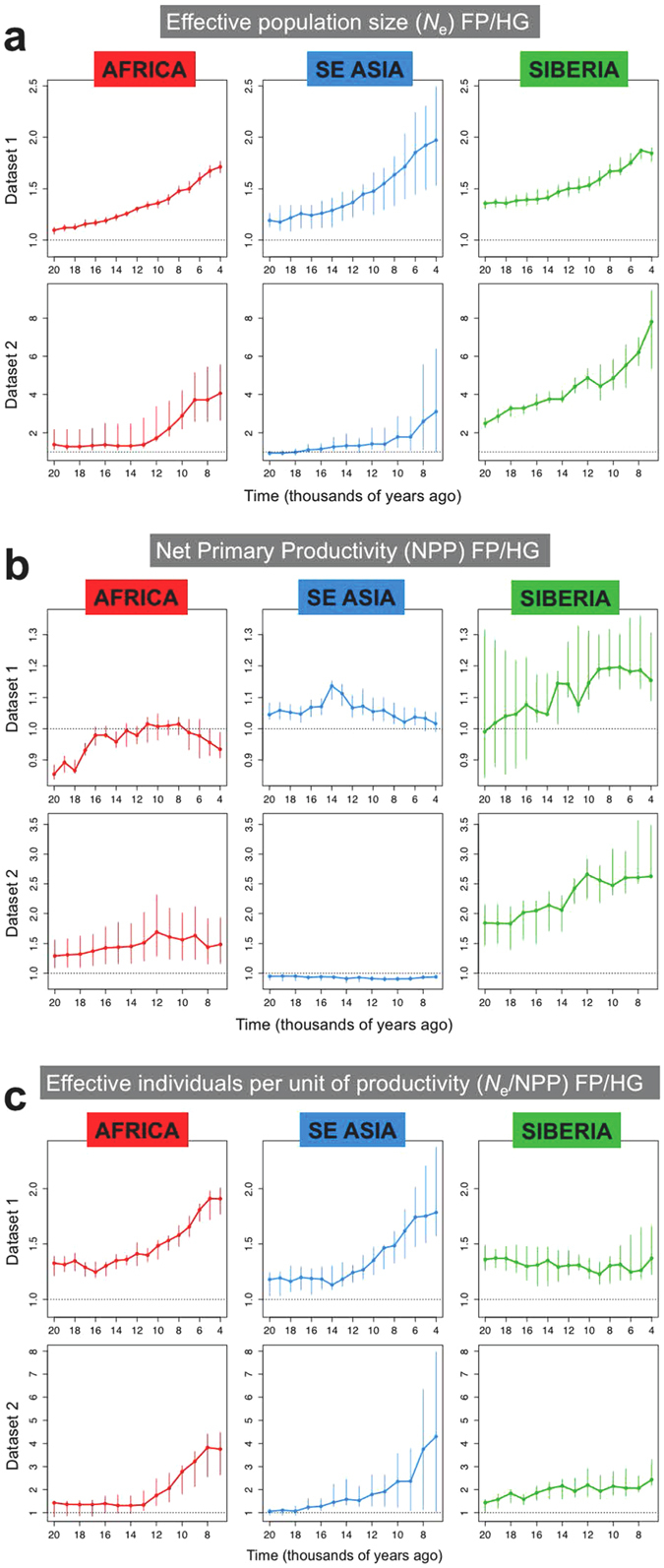
Median of the ratio of food producers over hunter-gatherers for *N*_e_ (**a**), NPP (**b**) and *N*_e_/NPP (**c**) in the three regions considered: sub-Saharan Africa, south-eastern Asia and Oceania, and Siberia. The plots show the temporal range between 4,000 and 20,000 years ago for dataset 1 and between 7,000 and 20,000 years ago for dataset 2. The error bars represent the 95% distribution of the jackknife leave-one-out validation.

For each region, we computed the ratio of population sizes among all pairs with a different subsistence regime (*N*_eFP_/*N*_eHG_, Fig. 2a) to investigate formally how hunter-gatherers and food producers differed through time: a ratio of 1 would indicate no significant differences between the two categories. For all three regions and both datasets, consistently food producers had larger populations than hunter-gatherers (minimum ratio = 1.1). This difference was already detectable 20k years ago (17 k years ago in SE Asia, dataset 2), i.e. long before the inception of food-producing activities, and increased through time. The detailed trajectories of each population are presented in the Supplementary Figures 1 and 2.

### Climate

We then tested whether these differences might be linked to climate. This explanation seems unlikely for Africa and SE Asia, as estimates of annual Net Primary Productivity (NPP) for these populations (assuming that they inhabited the same regions as in present times) were not consistently skewed in favor of future food producers (Fig. 2b). In Siberia, on the other hands, food producers inhabit areas that became progressively more favorable compared to those where hunter-gatherers are found. Indeed, if we quantify the number of effective individuals per unit of productivity (*N*_e_/NPP), we see that the ratio for food producers versus hunter-gatherers was above 1 well before the advent of food production (Fig. 2c). For Africa and SE Asia, this ratio increased markedly around 10–12 k years ago, implying either an increase in the number of effective individuals sustained by the same amount of resources, or some immigration, while for Siberia the ratio remained flat through time.

### Test for bottleneck in hunter-gatherers

The comparison between *NeON* trajectories calculated for American and European populations suggest that this method could underestimate *N*_e_ prior to a bottleneck^46^. Most modern-day hunter-gatherers are likely to have undergone repeated phenomena of fragmentation and/or demographic crisis, and, if so, our results could reflect to an extent that we cannot quantify a methodological bias. In other words, as previously observed^6^, based on measures of genetic diversity one may not be able to discriminate between long-term small population sizes, and recent bottlenecks affecting an originally large population.

MSMC is more robust to bottlenecks, and a comparison between the trajectories estimates with *NeON* and MSMC for the 12 populations that are shared between the two datasets shows that individual trajectories do indeed appear different when investigated by different methods, but this happens for all lifestyles, not only foragers (Supplementary Dataset 1), and the overall pattern when comparing lifestyles remains the same in both datasets.

### Migration

An important assumption of our approach is that the populations in our study lived at approximately the same location over the last 20 k years, thus discounting the possibility of long-distance migrations. We tested the effect of this assumption for SE Asia, where the population analyzed have been suggested to derive from at least two waves of advance^29,37–39^. The more recent dispersal, the so-called Austronesian expansion, is documented in the archaeological record. It is interpreted as a spread of food producers from continental eastern Asia associated with the diffusion of Neolithic cultures and technologies, starting between 6,000 and 4,000 years ago^2,40,41^. Two main routes have been proposed for it: under the “Fast train” model^42^ the expansion started from China and spread through Taiwan reaching then island South-East Asia and Oceania. The “Slow boat” scenario^43^, instead, postulates a spread from Mainland South-East Asia.

To take into account the mentioned hypotheses, we calculated the ratio of *N*_e_ in SE Asia Dataset 1 following three models: “Cultural diffusion” (absence of migration, as presented in the main results section), “Fast Train” and “Slow Boat”. Under the cultural diffusion model, we associated to each Austronesian population the NPP of the region where they are now living (as we had done in the previous analyses). Under the “Fast Train” and “Slow Boat” models, we associated to them the NPP of the source region, respectively China and Cambodia.

**Figure 3:**
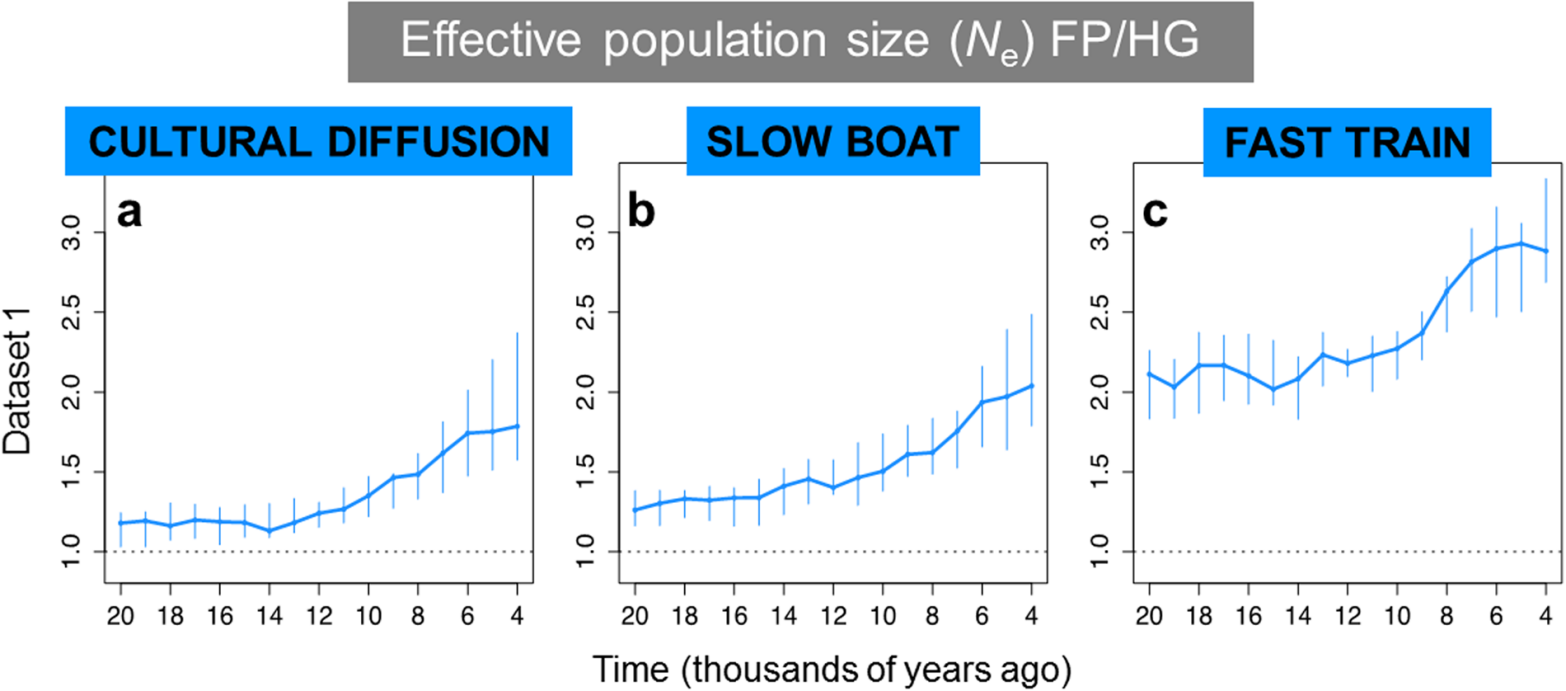
Median of the ratio of food producers over hunter-gatherers for *N*_e_/NPP following three different models of Neolithization of SE Asia and Oceania. (**a**) Cultural diffusion; (**b**) “Slow Boat” model (through Indonesia); (**c**) “Fast Train” model (Out of Taiwan).

As shown in Fig. 3, the overall pattern indicating an increase in more recent times does not vary much between models, showing that long distance migration does not seem to affect strongly our results. The main difference observed between models is that the minimum ratio is higher following the “Fast train” scenario. The reason is that the estimates of NPP for Eastern Asia are much lower than the ones observed in Taiwan and south-eastern Asia: as a consequence, the number of effective individuals per unit of NPP becomes much higher for food producers if based in a more temperate region such as China compared to more tropical areas.

## Discussion

Our global panel of populations revealed marked differences in inferred *N*_e_ between food producers and hunter-gatherers: as expected, the latter show larger effective population size. What was less obviously expected is that those differences began to accumulate 20 k years ago in all three regions, becoming more marked ~12–10 k years ago in Africa and SE Asia. It has to be stressed that these dates have been obtained using a generation time of 25 years^44^, which is considered an underestimate by some authors^45^, but allows direct comparisons with other studies. Had we chosen 30 years (e.g. as in ref.^37^), our time estimates for demographic growth would move even further back in time.

It is important to highlight that demographic estimates may suffer from different kinds of biases^6^. The method developed by McEvoy and colleagues appears to underestimate the *N*_e_ of non-Africans prior to the Out of Africa^35,39^, showing either a problem when dealing with bottlenecks (as suggested also by estimates on American populations^46^), or the confounding effect of population subdivision^47^. Even MSMC is not immune from the latter: the coalescent rate shows the same type of change when the population shrinks, and when a population of constant size gets subdivided^48^.

Neither possibility can be excluded, namely that future food producers already had larger population sizes before the inception of agriculture, or that hunter-gatherers have undergone repeated phenomena of fragmentation and/or demographic crisis. We tend to support the former view because, comparing estimates from different methods, it does not appear that only foragers have been subjected to such phenomena, possibly because most of the populations analysed are currently small and isolated groups of anthropological interest, rather than urban groups.

A certain level of uncertainty, at present, seems impossible to eliminate, and this is the reason why we prefer not to discuss the specific demographic reconstructions, but to focus instead on the comparison between lifestyles. Indeed, even if the individual trajectories may vary between *NeON* and MSMC, both datasets show the same signal of a difference between foragers and food producers that started before food production in agricultural communities. Moreover, our results match what has been already suggested based on other regions, markers and methods^11,13–19^, which makes us confident that the signal in the data is not dependent on the particular set of SNPs, or populations, or statistics chosen.

Whilst there are consistent differences between lifestyles across the three major regions covered by our study, Siberia stands out for showing a clear effect of climate. In this region, the increase in the difference between the two lifestyles can be mostly ascribed to food producers living in areas where the environment ameliorated markedly after the Last Glacial Maximum compared to more challenging conditions encountered by those populations that remained hunter-gatherers.

In other regions, on the other hand, we could not detect any effect of climate. We should emphasize that our reconstructions would only recover the medium-scale climatic conditions (in the order of 100 s of kilometers) that were encountered by a population, and would not be able to capture the effects of differences in the availability of local resources with a patchy distribution in space (such as freshwater from local rivers). Such local resources might well have played an important role for a number of populations; access to localized high-value resources has also been argued to develop societal structures that favor ownership and territory defense, and could have predisposed certain groups to be more likely to take up food production. On the other hand, the rough geographic resolution makes this method robust to short-distance migration, while the effects of long-distance migrations have been explicitly taken into account with the test performed on SE Asian populations, and is unlikely to account for the early dates of demographic growth inferred from the data.

The individual demographic trajectories in many cases show a decrease in the chronological window between 10 and 4 kya, corresponding to the establishment of food production in the analysed regions (Supplementary Figures 1 and 2), in contrast with archaeological data suggesting a demographic expansion following the Neolithic transition. Whilst the reasons for these declines remain unclear, they are in line with previous analyses, whether based on patterns of linkage disequilibrium on SNPchip data (e.g. ref.^35^), or on whole genomes analysed by PSMC (e.g. ref.^37^). The patterns found in our analysis seem then robust, as they are consistent with analyses based on different datasets and methods.

Apparent declines in *N*_e_ do not mean that the overall population was necessarily shrinking in size; indeed, the effective population size is affected by a variety of factors, including sex ratio, marriage patterns between and within groups, immigration, etc. Whatever the reason of this apparent decline could be, our analyses show that effective population sizes began to become larger in the ancestors of today’s food producers than in the ancestors of today’s foragers before the Neolithic transition (Fig. 2a). It seems more than likely that without the development of a new and, in the long run, more efficient subsistence technology, such an increase could not have lasted; agriculture doubtless created the resources to sustain larger populations. However, the demographic changes identified in our study cannot be regarded as a mere consequence of the increased food availability, but rather as a process preceding, and possibly stimulating, the Neolithic technological developments.

An early increase in *N*_e_ that predates Neolithization has been interpreted as capturing early societal changes that might have favored the later development of food production^49^. High population density can facilitate technical innovation, and populations in more advantageous areas that sustained higher densities might have led to the later improvement in subsistence technologies^50^. Moreover, in modern-day hunter-gatherers a larger population relative to ecological productivity is positively correlated to complex behaviors such as sedentism, storage activity and social stratification^51^.

Furthermore, indirect estimates of *N*_e_ from genetic data can also reflect immigration to an extent that can hardly be predicted, (with migration among previously isolated populations increasing *N*_e_); areas where movement among populations and more connected networks of potential innovators might favor the development of food production in a manner similar to larger overall populations. Thus, large estimated *N*_e_ values might not represent just a large census size, but also high gene flow (and hence cultural connectivity), both of which could have favored innovation.

These two mechanisms are not mutually exclusive, and it is difficult to disentangle them genetically. However, the key result from our analysis is that, even when using a combination of genomes and a large amount of genome-wide data from a globally-distributed panel of populations, populations that later adopted food productions differed from those who remained hunter-gatherers well before their lifestyle changed. This process did not happen as result of differential resources but because of cultural, behavioral or social causes, maybe the same that have led to the major population replacement in Europe when hunter-gatherers and farmers met^27,52–54^. The very limited number of modern-day foragers from Western Eurasia, and the lack of genetic data from them, do not allow a direct test with the approach presented here, but similar results on European populations have been obtained with other methods^19^. Therefore, we conclude that pre-existing cultural or demographic differences among Paleolithic hunter-gatherers in the Old World likely played a role in the later choice of adopting food production.

## Materials and Methods

### Datasets

**Dataset 1**: We compiled an extensive dataset of publicly available SNP data. We analyzed populations of hunter-gatherers and food producers from three regions in which both lifestyles are present in modern times (Fig. 1): sub-Saharan Africa (28 populations), southeastern Asia and Oceania (17 populations), Siberia (19 populations) (more information can be found in Supplementary Table 1). Only populations with a minimum of 15 individuals (10 for south-eastern Asia and Oceania) were considered, giving a total of >1200 individuals. The minimum number of SNPs used for the analyses for any given population was 150 k.

**Dataset 2**: MSMC demographic estimates for a large panel of worldwide populations have been published in the supplementary material of a recent publication by Pagani and colleagues^37^. The choice of populations is more limited, but populations with different lifestyles were available for all the three regions where SNP data were collected: Africa (5 populations) SE Asia (9 populations) and Siberia (10 populations) (Supplementary Table 2).

Lifestyle information for each population, when not available in the original reference, was recovered from Levinson (1991)^55^. Many Siberian populations adopt a variety of subsistence strategies, and in those cases, we classified them based on their primary activity. Geographic locations, when not available in the original references, where calculated as the center or the capital of the country where the sampling has been performed.

### Estimation of *N*_e_ through time for Dataset 1

For each population, we used the pattern of Linkage Disequilibrium (LD) to estimate changes in *N*_e_ through time using the approach by McEvoy *et al*.^35^ as implemented in the R package *NeON*^46^. Given a known recombination rate, the amount of linkage disequilibrium (LD) between differently spaced loci can help reconstruct past values of *N*_e_^56^. The reason is that smaller *N*_e_ leads to higher genetic drift, and hence to increased LD values. However, the greater the recombination rate between pairs of genetic markers, the faster the decay of LD between them. Since recombination accumulates through time, LD over large recombination distances gives an estimate of *N*_e_ in recent times, while LD over short recombination distances is informative on ancient *N*_e_^57^.

*NeON* calculates the recombination rates for each possible pair of markers taking their genetic distance into account. We retrieved genetic maps of the human genome from the HapMap website (https://www.ncbi.nlm.nih.gov/probe/docs/projhapmap/), and the SNPs available for each population were mapped accordingly. Markers which could not be located on the HapMap maps were discarded.

In *NeON*, estimates of *N*_e_ are obtained by first assigning pairs of markers into several classes as a function of the recombination distance between them, and then calculating the squared correlation coefficient of linkage disequilibrium (r_2_^LD^)^58^. The r_2_^LD^ is then used to estimate the value of effective population size within each of the identified categories, which, as discussed above, corresponds to the effective population size at a specific moment in the past.

### Comparing hunter-gatherers and foragers

For both datasets we summarized the demographic estimates by computing the harmonic mean of *N*_e_ every 1,000 years from 20,000 until 4,000 years ago, using a generation time of 25 years^44^ (while in the original publication for Dataset 2 they use a generation time of 30 years)^37^. Given the low number of foragers in dataset 2, we considered as foragers not only hunter-gatherers but also horticulturalists, that in the analyses of dataset 1 are considered food producers.

For each region, we calculated the ratio between values of *N*_e_ in each possible pair of populations with different lifestyles (food producers over foragers, *N*_eFP_/*N*_eHG_). We then plotted the median of the ratio and calculated the error as the 95% distribution of the jackknife leave-one-out validation. A ratio of 1 would mean that the two different lifestyles have, on average, the same *N*_e_.

Differences in *N*_e_ could be linked to a variety of environmental factors, such as climate and environmental productivity. To quantify this effect, we extracted Net Primary Productivity (NPP) estimates from paleoclimatic reconstructions^59^. We explored the changes in resource availability between populations with difference lifestyles by plotting with the same method described above for *N*_e_ the ratio between the estimates of NPP for the two lifestyles considered (NPP_FP_/NPP_HG_).

Finally, to correct for environmental effects on effective population size, we normalised *N*_e_ by NPP (number of effective individuals per unit of primary productivity). We again used the same method to calculate the median and 95% CI of *N*_e_/NPP for food producers over hunter-gatherers. A ratio of 1 would mean that, once corrected for NPP, populations with different subsistence use the natural resources with the same efficiency.

### Test of the method: Integrating long-distance migration in SE Asia

The approach employed in this paper ignores the effect of long distance migrations. This assumption is clearly unrealistic. To test how much the results could change if such migrations were taken into account, we modelled the Austronesian expansion in south-eastern Asia and Oceania under both the “Fast train”^42^ and the “Slow Boat”^43^ models. We then compared the results with the ones issued under the “Cultural Diffusion” model (absence of population movements).

For each model, *N*_e_/NPP was calculated in a different way. Under the cultural diffusion model, we associated to each Austronesian population the NPP of the region where they are now living (the same logic used in the earlier analyses). Under the “Fast Train” and “Slow Boat” models, we associated to them NPP of the source region, respectively China and Cambodia.

Also in sub-Saharan Africa, there is evidence of a massive migration, the so-called “Bantu expansion”. However, since only one out of 28 African populations in our dataset belongs to the Bantu, this migration would have had a negligible effect on the analyses presented in this paper.

## Electronic supplementary material

Supplementary file
