# Author Correction: An earlier revolution: genetic and genomic analyses reveal pre-existing cultural differences leading to Neolithization

**DOI:** 10.1038/s41598-017-18588-0

**Published:** 2018-01-09

**Authors:** Michela Leonardi, Guido Barbujani, Andrea Manica

**Affiliations:** 1 Department of Life Sciences and Biotechnology, University of Ferrara, Via Borsari 44, 44121 Ferrara, Italy; 2 Department of Zoology, University of Cambridge, Downing street, CB2 3EJ Cambridge, UK; 3 Present Address: Centre for GeoGenetics, Natural History Museum of Denmark, University of Copenhagen, Oester Voldgade 5-7, DK-1350 Copenhagen, Denmark

Correction to: *Scientific Reports* 10.1038/s41598-017-03717-6, published online 14 June 2017

In Figure 3, the title ‘Effective individuals per unit of productivity (Ne/NPP) FP/HG’ is incorrectly given as ‘Effective population size (*N*_e_) FP/HG’. The correct Figure 3 appears below as Figure 1.

This Article also contains errors in the Results section under subheading ‘Migration’.

“To take into account the mentioned hypotheses, we calculated the ratio of *N*_e_ in SE Asia Dataset 1”

should read:

“To take into account the mentioned hypotheses, we calculated the ratio of *N*_e_ /NPP for food producers over foragers in SE Asia Dataset 1”

**Figure 1:**
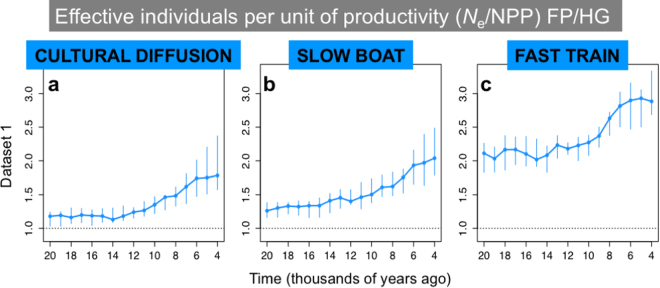
.

