# Supplementary material for: An earlier revolution: genetic and genomic analyses reveal pre-existing cultural differences leading to Neolithization
Source: Sci Rep. 2017 Jun 14;7:3525. doi: 10.1038/s41598-017-03717-6 (PMC5471218; doi:10.1038/s41598-017-03717-6)
Supplement: Supplementary file 1 — Supplementary file [file 41598_2017_3717_MOESM1_ESM.doc]

**Title:** An earlier revolution: genetic and genomic analyses reveal pre-existing cultural differences leading to Neolithization.

**Authors:** Michela Leonardi* ^a,b^ Guido Barbujani ^a^, Andrea Manica ^c^

**Affiliations:**

^a^ Department of Life Sciences and Biotechnology, University of Ferrara, Via Borsari 44, 44121 Ferrara, Italy

^b^ current position: Centre for GeoGenetics, Natural History Museum of Denmark, University of Copenhagen, Oester Voldgade 5-7, DK-1350 Copenhagen, Denmark

^c^ Department of Zoology, University of Cambridge, Downing street, CB2 3EJ, Cambridge, UK.

*Corresponding Author: Michela Leonardi, Centre for GeoGenetics, Natural History Museum of Denmark, University of Copenhagen, Oester Voldgade 5-7, DK-1350 Copenhagen, Denmark. Phone number: 0045 50 30 77 42. Email: michela.leonardi@smn.ku.dk

**Orcid ID:**

ML: 0000-0001-8933-9374

GB: 0000-0001-7854-6669

AM: 0000-0003-1895-450X

## Supplementary material

**Supplementary table 1**: List of populations and associated information for Dataset 1. In the last column shows the final number of SNPs used for the analyses. Chukchi and Chukchi L represent two different sets of SNPs from the same population, they were kept separated because the number of overlapping SNPs was low.

| **Region** | **Population** | **Total** | **Country of**  **origin** | **Lifestyle** | **reference** | **SNPs** |
| --- | --- | --- | --- | --- | --- | --- |
| Sub-saharan Africa | Amhara | 26 | Ethiopia | Agriculturalists | (1) | 832 854 |
| Sub-saharan Africa | Anuak | 23 | Ethiopia | Mixed farming | (1) | 832 854 |
| Sub-saharan Africa | Ari Blacksmith | 17 | Ethiopia | Agriculturalists | (1) | 832 854 |
| Sub-saharan Africa | Ari Cultivator | 24 | Ethiopia | Agriculturalists | (1) | 832 854 |
| Sub-saharan Africa | Bamoun | 20 | Cameroon | Agriculturalists | (2) | 342 309 |
| Sub-saharan Africa | Biaka | 23 | CAR | Hunter gatherers | (3) | 324 881 |
| Sub-saharan Africa | Bulala | 15 | Chad | Agriculturalists | (2) | 342 309 |
| Sub-saharan Africa | Ethiopian Somali | 17 | Ethiopia | Pastoralists | (1) | 832 854 |
| Sub-saharan Africa | Fang | 18 | Cameroon | Agriculturalists | (2) | 342 309 |
| Sub-saharan Africa | Gui and Ghana | 15 | Botswana | Hunter gatherers | (4) | 985 001 |
| Sub-saharan Africa | Gumuz | 19 | Ethiopia | Pastoralists | (1) | 832 854 |
| Sub-saharan Africa | Hadza | 17 | Tanzania | Hunter gatherers | (5) | 519 770 |
| Sub-saharan Africa | Igbo | 17 | Nigeria | Agriculturalists | (2) | 342 309 |
| Sub-saharan Africa | Juhoansi | 18 | Namibia | Hunter gatherers | (4) | 985 001 |
| Sub-saharan Africa | Kaba | 16 | Chad | Agriculturalists | (2) | 342 309 |
| Sub-saharan Africa | Karretjie | 20 | South Africa | Hunter gatherers/Pastoralists | (4) | 985 001 |
| Sub-saharan Africa | Khomani | 66 | South Africa | Hunter gatherers | (4), (5) | 290 516 |
| Sub-saharan Africa | Khwe | 17 | Angola, Namibia | Hunter gatherers | (4) | 985 001 |
| Sub-saharan Africa | Mandenka | 22 | Senegal | Agriculturalists | (3) | 324 881 |
| Sub-saharan Africa | Nama | 20 | Namibia | Pastoralists | (4) | 985 001 |
| Sub-saharan Africa | Oromo | 21 | Ethiopia | Agriculturalists/Pastoralists | (1) | 832 854 |
| Sub-saharan Africa | Sandawe | 28 | Tanzania | Hunter gatherers | (5) | 519 770 |
| Sub-saharan Africa | South African Bantu | 20 | South Africa | Pastoralists | (4) | 985 001 |
| Sub-saharan Africa | Somali | 23 | Somalia | Agriculturalists | (1) | 832 854 |
| Sub-saharan Africa | South Sudanese | 24 | Sudan | Agriculturalists | (1) | 832 854 |
| Sub-saharan Africa | Tigray | 21 | Ethiopia | Agriculturalists | (1) | 832 854 |
| Sub-saharan Africa | Xun | 19 | Angola | Hunter gatherers | (4) | 985 001 |
| Sub-saharan Africa | Yoruba | 22 | Nigeria | Agriculturalist | (3) | 324 881 |
| South-Eastern Asia and Oceania | Aboriginal Australian | 12 | Australia | Hunter-gatherers | (10) | 260 660 |
| South-Eastern Asia and Oceania | Aeta | 37 | Philippines | Hunter-gatherers | (11) | 164 690 |
| South-Eastern Asia and Oceania | Burmese | 15 | Myanmar | Agriculturalists | (12) | 522 180 |
| South-Eastern Asia and Oceania | Borneo | 39 | Indonesia | Agriculturalists | (10) | 200 403 |
| South-Eastern Asia and Oceania | Cambodians | 10 | Cambodia | Agriculturalists | (3) | 324 881 |
| South-Eastern Asia and Oceania | Fiji | 25 | Fiji | Horticulturalists | (10) | 200 403 |
| South-Eastern Asia and Oceania | Mamanwa | 11 | Philippines | Hunter-gatherers | (10) | 200 403 |
| South-Eastern Asia and Oceania | Manobo | 16 | Philippines | Agriculturalists | (10) | 200 403 |
| South-Eastern Asia and Oceania | Moluccas | 10 | Indonesia | Agriculturalists | (10) | 200 403 |
| South-Eastern Asia and Oceania | Nusa Tengarras | 10 | Indonesia | Agriculturalists | (10) | 200 403 |
| South-Eastern Asia and Oceania | Papuans | 17 | Papua New Guinea | Horticulturalists | (3) | 324 881 |
| South-Eastern Asia and Oceania | Onge | 10 | India | Hunter-gatherers | (13) | 200 403 |
| South-Eastern Asia and Oceania | PNG highlanders | 24 | Papua New Guinea | Horticulturalists | (10) | 200 403 |
| South-Eastern Asia and Oceania | Polynesians | 19 | Polynesia | Horticulturalists | (10) | 200 403 |
| South-Eastern Asia and Oceania | Sumatra | 17 | Sumatra | Agriculturalists | (10) | 200 403 |
| South-Eastern Asia and Oceania | Taiwan Aborigines | 12 | Taiwan | Hunter-gatherers | (10) | 200 403 |
| South-Eastern Asia and Oceania | Temuan | 10 | Malaysia | Agriculturalists | (10) | 200 403 |
| Siberia and Russia | Altaians | 19 | Russia | Pastoralists | (14), (15), (16) | 300 708 |
| Siberia and Russia | Buriats | 22 | Russia | Pastoralists | (15), (16) | 308 416 |
| Siberia and Russia | Chukchi | 17 | Russia | Hunter-gatherers | (15), (16), (17) | 307 170 |
| Siberia and Russia | Chukchi L | 23 | Russia | Hunter-gatherers | (6) | 318 241 |
| Siberia and Russia | Russians (Central) | 32 | Russia | Agriculturalists | (15) | 308 786 |
| Siberia and Russia | Eskimo | 22 | Russia | Hunter-gatherers | (6) | 318 241 |
| Siberia and Russia | Evenki | 19 | Russia | Hunter gatherers | (15), (16) | 308 416 |
| Siberia and Russia | Khakas | 17 | Russia | Pastoralists | (17) | 534 413 |
| Siberia and Russia | Koryak | 20 | Russia | Hunter-gatherers | (15), (16), (17) | 307 170 |
| Siberia and Russia | Nganassan | 17 | Russia | Hunter-gatherers | (15), (16) | 308 416 |
| Siberia and Russia | Russians (North) | 25 | Russia | Agriculturalists | (3) | 164 690 |
| Siberia and Russia | Selkup | 17 | Russia | Hunter-gatherers | (14), (16) | 518 962 |
| Siberia and Russia | Siberian Tatars | 20 | Russia | Agriculturalists | (15) | 308 786 |
| Siberia and Russia | Tubalar | 22 | Russia | Pastoralists | (6) | 318 241 |
| Siberia and Russia | Tuvans | 19 | Russia | Pastoralists | (15), (16) | 308 416 |
| Siberia and Russia | Udmurt | 16 | Russia | Agriculturalists | (15) | 308 786 |
| Siberia and Russia | Ulchi | 25 | Russia | Hunter-gatherers | (6) | 318 241 |
| Siberia and Russia | Yakut | 25 | Russia | Pastoralists | (3) | 324 881 |
| Siberia and Russia | Yukagir | 19 | Russia | Hunter-gatherers | (6) | 318 241 |

**Supplementary table 2**: List of populations and associated information for Dataset 2, from (18).

| **Region** | **Population** | **Country of origin** | **Lifestyle** |
| --- | --- | --- | --- |
| Sub-Saharan Africa | Baka-Pygmies | Cameroon | Hunter-gatherers |
| Sub-Saharan Africa | Hadza | Tanzania | Hunter-gatherers |
| Sub-Saharan Africa | Luhya | Kenya | Agriculturalists |
| Sub-Saharan Africa | Sandawe | Tanzania | Hunter-gatherers |
| Sub-Saharan Africa | Yoruba | Nigeria | Agriculturalists |
| Siberia and Russia | Altaians | Russia | Agriculturalists |
| Siberia and Russia | Udmurds | Russia | Agriculturalists |
| Siberia and Russia | Maris | Russia | Agriculturalists |
| Siberia and Russia | Vepsas | Russia | Agriculturalists |
| Siberia and Russia | Bashkirs | Russia | Agriculturalists |
| Siberia and Russia | Buryats | Russia | Agriculturalists |
| Siberia and Russia | Mongolians | Mongolia | Pastoralists |
| Siberia and Russia | Chukchis | Russia | Hunter-gatherers |
| Siberia and Russia | Eskimo | Russia | Hunter-gatherers |
| Siberia and Russia | Koryaks | Russia | Hunter-gatherers |
| Siberia and Russia | Yakuts | Russia | Pastoralists |
| South-Eastern Asia and Oceania | Bajo | Indonesia | Hunter-gatherers |
| South-Eastern Asia and Oceania | Dusun | Brunei | Agriculturalists |
| South-Eastern Asia and Oceania | Igorot | Philippines | Agriculturalists |
| South-Eastern Asia and Oceania | Lebbo | Indonesia | Horticulturalists |
| South-Eastern Asia and Oceania | Murut | Brunei | Agriculturalists |
| South-Eastern Asia and Oceania | Burmese | Myanmar | Agriculturalists |
| South-Eastern Asia and Oceania | Vietnamese_north | Vietnam | Agriculturalists |
| South-Eastern Asia and Oceania | Vietnamese_south | Vietnam | Agriculturalists |
| South-Eastern Asia and Oceania | Koinanbe | Papua New Guinea | Horticulturalists |

**Supplementary Figure 1:** Variation of the effective population size (*N*e) and Net Primary Productivity (NPP) through time for each population of Dataset 1 in the three regions considered. Black lines represent hunter gatherers while colored lines represent food producer populations.


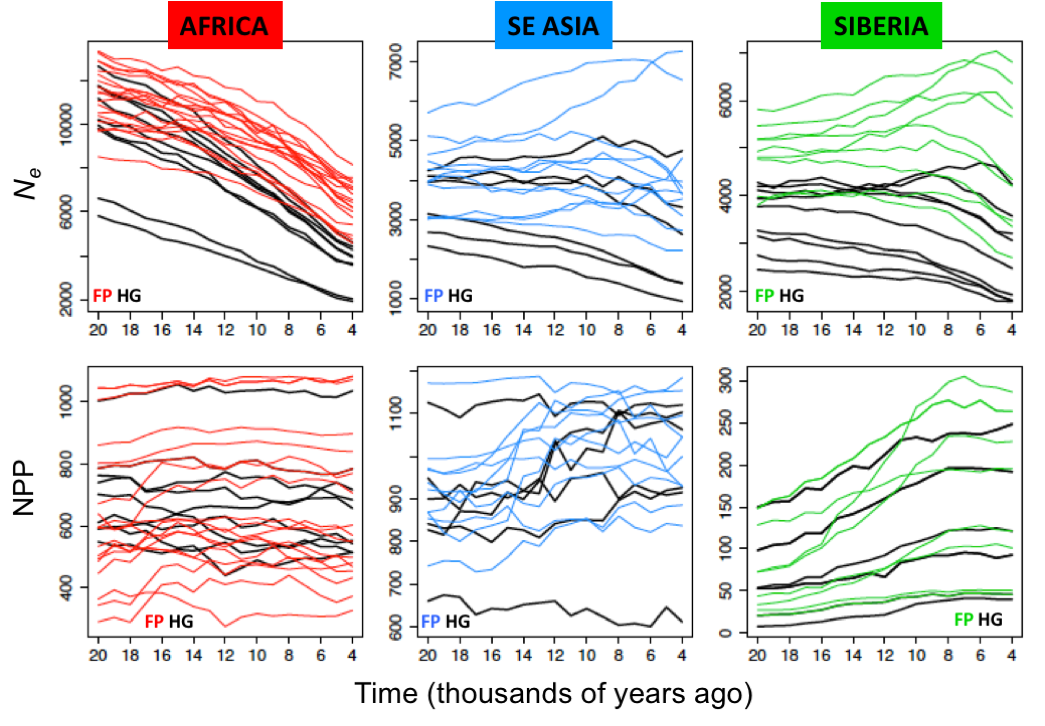


**Supplementary Figure 2**: Variation of the effective population size (*N*e) and Net Primary Productivity (NPP) through time for each population of Dataset 2 in the three regions considered. Black lines represent hunter gatherers while colored lines represent food producer populations.

**
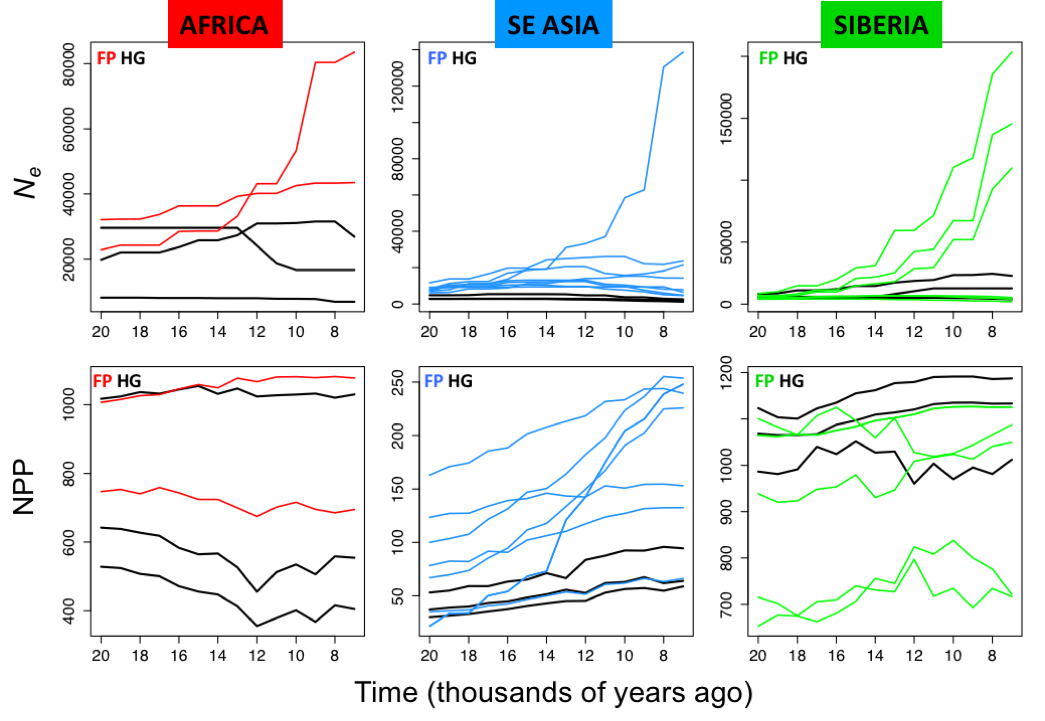
**

**Supplementary Table 3**: comparison between *NeON* and MSMC *N*_e_ estimates for population shared between Dataset 1 and 2. The MSMC estimates (Pagani et al. 2016, Dataset 2 in this file) have been corrected for a generation time of 25 years instead of 30, as presented in the original publication.

| Region | Lifestyle | Population | 4,000 ya | 5,000 ya | 6,000 ya | 7,000 ya | 8,000 ya | 9,000 ya | 10,000 ya | 11,000 ya | 12,000 ya | 13,000 ya | 14,000 ya | 15,000 ya | 16,000 ya | 17,000 ya | 18,000 ya | 19,000 ya | 20,000 ya |
| --- | --- | --- | --- | --- | --- | --- | --- | --- | --- | --- | --- | --- | --- | --- | --- | --- | --- | --- | --- |
| SEAsia | Agriculturalists | Burmese dataset 1 | 6544 | 6716 | 7020 | 7058 | 7042 | 6999 | 6969 | 6761 | 6693 | 6593 | 6478 | 6286 | 6071 | 5904 | 5972 | 5890 | 5711 |
|  |  | Burmese dataset 2 | 74949316 | 1429807 | 951667 | 203337 | 185430 | 117984 | 110301 | 71572 | 59355 | 59355 | 30945 | 29156 | 19977 | 14820 | 14820 | 9968 | 8495 |
| Siberia | Agriculturalists | Udmurts dataset 1 | 3480 | 3619 | 3962 | 4252 | 4392 | 4439 | 4543 | 4511 | 4618 | 4608 | 4682 | 4734 | 4687 | 4770 | 4687 | 4751 | 4762 |
|  |  | Udmurts dataset 2 | 5876 | 4410 | 3617 | 4691 | 5160 | 6657 | 7621 | 8278 | 9705 | 9705 | 10303 | 10577 | 10360 | 9827 | 9827 | 9212 | 8239 |
| Africa | Agriculturalists | Yoruba dataset 1 | 7571 | 7582 | 8340 | 8921 | 9208 | 9431 | 9826 | 10456 | 10486 | 10657 | 10747 | 10921 | 11224 | 11465 | 11647 | 11235 | 11516 |
|  |  | Yoruba dataset 2 | 2765276 | 87253 | 84524 | 83511 | 80363 | 80363 | 53231 | 43146 | 43146 | 33147 | 28610 | 28610 | 28482 | 24254 | 24254 | 24254 | 22860 |
| Siberia | Pastoralists | Altaians dataset 1 | 5660 | 5852 | 6110 | 6157 | 6103 | 5999 | 6099 | 5957 | 5987 | 5907 | 5754 | 5613 | 5570 | 5545 | 5523 | 5489 | 5469 |
|  |  | Altaians dataset 2 | 20005 | 25584 | 37478 | 21472 | 18192 | 16594 | 15763 | 16863 | 20491 | 20491 | 19240 | 18528 | 17042 | 12367 | 12367 | 11261 | 8186 |
| Siberia | Pastoralists | Buryat dataset 1 | 4206 | 4432 | 4726 | 4889 | 4980 | 5169 | 4946 | 5028 | 4942 | 4984 | 4953 | 4943 | 4922 | 4848 | 4815 | 4806 | 4808 |
|  |  | Buryat dataset 2 | 13802 | 11992 | 11589 | 14279 | 14332 | 15323 | 15342 | 14205 | 14019 | 12710 | 11184 | 11184 | 8813 | 8302 | 8302 | 6356 | 6167 |
| Siberia | Pastoralists | Yakut dataset 1 | 3355 | 3619 | 3858 | 3893 | 4025 | 4076 | 4048 | 3967 | 4075 | 4111 | 3985 | 4021 | 3996 | 4039 | 4063 | 4003 | 3804 |
|  |  | Yakut dataset 2 | - | - | 5613 | 6422 | 9403 | 9403 | 9403 | 9403 | 9403 | 9403 | 9403 | 9403 | 9217 | 9181 | 9181 | 9181 | 9181 |
| Siberia | Hunter-gatherers | Koryak dataset 1 | 1805 | 1934 | 2137 | 2269 | 2413 | 2570 | 2653 | 2740 | 2775 | 2811 | 2922 | 2896 | 2954 | 3010 | 3105 | 3063 | 3157 |
|  |  | Koryak dataset 2 | 1022 | 1236 | 1671 | 1828 | 2302 | 2364 | 2654 | 2742 | 2878 | 2881 | 2981 | 2981 | 2938 | 2929 | 2927 | 2881 | 2881 |
| Siberia | Hunter-gatherers | Chukchi dataset 1 | 2485 | 2655 | 2827 | 3028 | 3119 | 3259 | 3379 | 3386 | 3486 | 3606 | 3663 | 3658 | 3725 | 3698 | 3783 | 3787 | 3776 |
|  |  | Chukchi L dataset 1 | 1819 | 1928 | 2109 | 2178 | 2289 | 2356 | 2400 | 2451 | 2466 | 2425 | 2501 | 2546 | 2616 | 2646 | 2636 | 2659 | 2759 |
|  |  | Chukchi dataset 2 | 1690 | 1876 | 2014 | 2582 | 2927 | 3706 | 3744 | 4789 | 4789 | 5353 | 5419 | 5428 | 5441 | 5441 | 4932 | 4852 | 4852 |
| Siberia | Hunter-gatherers | Eskimo dataset 1 | 1795 | 1804 | 1948 | 2053 | 2190 | 2236 | 2293 | 2252 | 2288 | 2323 | 2307 | 2356 | 2386 | 2425 | 2418 | 2428 | 2458 |
|  |  | Eskimo dataset 2 | 1029 | 1033 | 1180 | 1236 | 1548 | 1702 | 1984 | 2268 | 2437 | 2544 | 2776 | 2776 | 2796 | 2798 | 2804 | 2815 | 2815 |
| Africa | Hunter-gatherers | Hadza dataset 1 | 2016 | 2196 | 2475 | 2778 | 3118 | 3507 | 3722 | 4201 | 4421 | 4718 | 5130 | 5357 | 5548 | 5708 | 6062 | 6428 | 6618 |
|  |  | Hadza dataset 2 | 4251 | 4831 | 5624 | 6939 | 6939 | 7794 | 7849 | 7869 | 8036 | 8036 | 8048 | 8110 | 8110 | 8118 | 8205 | 8205 | 8205 |
| Africa | Hunter-gatherers | Biaka dataset 1 | 3637 | 3739 | 4324 | 4997 | 5284 | 5897 | 6200 | 6691 | 6969 | 7781 | 8125 | 8646 | 8704 | 9151 | 9240 | 9458 | 9977 |
|  |  | Biaka dataset 2 | - | - | 16655 | 16655 | 16655 | 16655 | 16655 | 18698 | 24144 | 29590 | 29590 | 29590 | 29590 | 29590 | 29590 | 29590 | 29590 |
| Africa | Hunter-gatherers | Sandawe dataset 1 | 4268 | 4656 | 5221 | 5743 | 6220 | 6860 | 7145 | 7713 | 8000 | 8375 | 8609 | 8860 | 9188 | 9452 | 9930 | 9937 | 10228 |
|  |  | Sandawe dataset 2 | 17115 | 24135 | 24135 | 26890 | 31516 | 31516 | 31048 | 30913 | 30913 | 27325 | 25759 | 25759 | 23671 | 22054 | 22054 | 22054 | 19777 |

**Supplementary figure 3**: comparison between *NeON* and MSMC *N*_e_ estimates for population shared between Dataset 1 (darker colors) and 2 (lighter colors).


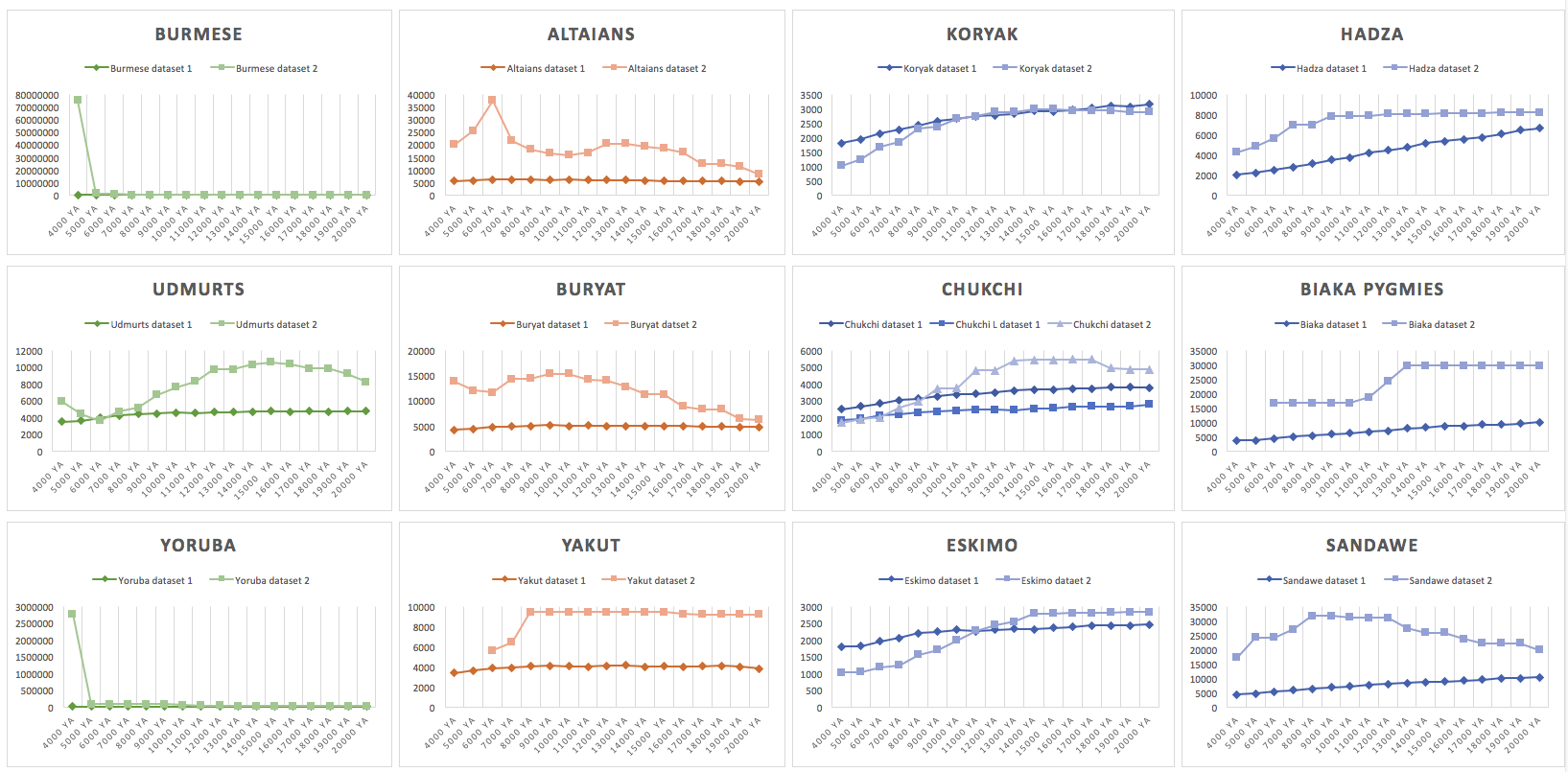


## References

1. Pagani L, et al. (2012) Ethiopian genetic diversity reveals linguistic stratification and complex influences on the Ethiopian gene pool. *Am J Hum Genet* 91(1):83–96.

2. Bryc K, et al. (2010) Genome-wide patterns of population structure and admixture in West Africans and African Americans. *Proc Natl Acad Sci U S A* 107(2):786–91.

3. Li JZ, et al. (2008) Worldwide human relationships inferred from genome-wide patterns of variation. *Science* 319(5866):1100–4.

4. Schlebusch CM, et al. (2012) Genomic variation in seven Khoe-San groups reveals adaptation and complex African history. *Science* 338(6105):374–9.

5. Henn BM, et al. (2011) Hunter-gatherer genomic diversity suggests a southern African origin for modern humans. *Proc Natl Acad Sci U S A* 108(13):5154–62.

6. Lazaridis I, et al. (2014) Ancient human genomes suggest three ancestral populations for present-day Europeans. *Nature* 513(7518):409–413.

7. Behar DM, et al. (2010) The genome-wide structure of the Jewish people. *Nature* 466(7303):238–42.

8. Haber M, et al. (2011) Influences of history, geography, and religion on genetic structure: the Maronites in Lebanon. *Eur J Hum Genet* 19(3):334–40.

9. Henn BM, et al. (2012) Genomic ancestry of North Africans supports back-to-Africa migrations. *PLoS Genet* 8(1):e1002397.

10. Reich D, et al. (2011) Denisova admixture and the first modern human dispersals into Southeast Asia and Oceania. *Am J Hum Genet* 89(4):516–528.

11. Rasmussen M, et al. (2011) An Aboriginal Australian genome reveals separate human dispersals into Asia. *Science* 334(6052):94–8.

12. Chaubey G, et al. (2011) Population genetic structure in Indian Austroasiatic speakers: the role of landscape barriers and sex-specific admixture. *Mol Biol Evol* 28(2):1013–24.

13. Reich D, Thangaraj K, Patterson N, Price AL, Singh L (2009) Reconstructing Indian population history. *Nature* 461(7263):489–94.

14. Raghavan M, et al. (2014) Upper Palaeolithic Siberian genome reveals dual ancestry of Native Americans. *Nature* 505(7481):87–91.

15. Yunusbayev B, et al. (2015) The genetic legacy of the expansion of Turkic-speaking nomads across Eurasia. *PLoS Genet* 11(4):e1005068.

16. Rasmussen M, et al. (2010) Ancient human genome sequence of an extinct Palaeo-Eskimo. *Nature* 463(7282):757–62.

17. Fedorova SA, et al. (2013) Autosomal and uniparental portraits of the native populations of Sakha (Yakutia): implications for the peopling of Northeast Eurasia. *BMC Evol Biol* 13:127.

18. Pagani L, et al. (2016) Genomic analyses inform on migration events during the peopling of Eurasia. *Nature* 538(7624):238–242.
